# Di­aqua­bis­(ethyl­enedi­amine-κ^2^ *N*,*N*′)copper(II) bis­(sulfamerazinate)

**DOI:** 10.1107/S160053681401068X

**Published:** 2014-05-17

**Authors:** Amani Direm, Wahiba Falek, Guillaume Pilet, Nourredine Benali-Cherif

**Affiliations:** a Laboratoire des Structures, Propriétés et Interactions Interatomiques, LASPI2A, Université "Abbes Laghrour", Khenchela 40.000, Algeria; b Université de Lyon, Laboratoire des Multimatériaux et Interfaces (LMI) UMR, 5615 CNRS Université Claude Bernard Lyon 1, Avenue du 11 Novembre 1918, 69622 Villeurbanne Cedex, France

## Abstract

The asymmetric unit of the title compound, [Cu(C_2_H_8_N_2_)_2_(H_2_O)_2_](C_11_H_11_N_4_O_2_S)_2_, contains one sulfamerazinate anion in a general position and one half-cation that is located on a center of inversion. The Cu^II^ cation shows a strong Jahn–Teller distortion. It is coordinated by four N atoms of two ethyl­enedi­amine ligands in the basal plane and two O atoms at much longer distances in the axial positions in a bipyramidal coordination. In the crystal, the building blocks are connected by N—H⋯N, O—H⋯N, N—H⋯O and O—H⋯O hydrogen bonding into a two-dimensional network parallel to (001).

## Related literature   

For the anti­bacterial activity of sulfonamides, see: Anand (1980 ▶); Kratz *et al.* (2000 ▶); Grave *et al.* (2010 ▶). For uses of sulfamerazine, see: Murphy *et al.* (1943 ▶); Clark *et al.* (1943 ▶); Earle (1944 ▶); Forbes *et al.* (1946 ▶). The crystal structure of sulfamerazine was reported by Acharya *et al.* (1982 ▶). For a related compound in which sulfa­thia­zole acts as a deproton­ated counter-ion, see: Anacona *et al.* (2002 ▶).
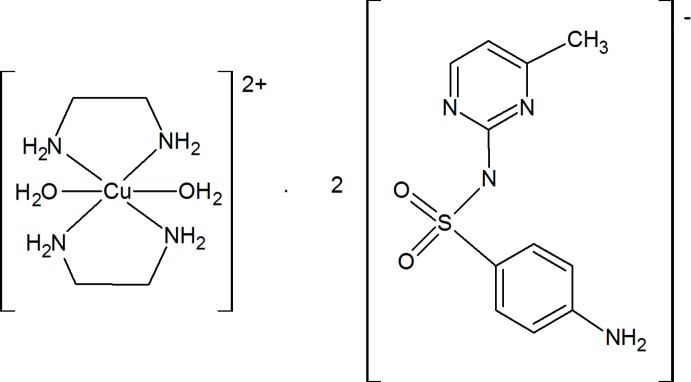


## Experimental   

### 

#### Crystal data  

[Cu(C_2_H_8_N_2_)_2_(H_2_O)_2_](C_11_H_11_N_4_O_2_S)_2_
*M* *_r_* = 746.41
Triclinic, 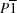

*a* = 7.5429 (4) Å
*b* = 8.1800 (5) Å
*c* = 14.8434 (8) Å
α = 75.299 (5)°
β = 82.800 (5)°
γ = 78.873 (5)°
*V* = 866.40 (9) Å^3^
*Z* = 1
Mo *K*α radiation
μ = 0.81 mm^−1^
*T* = 293 K
0.41 × 0.36 × 0.17 mm

#### Data collection  

Oxford Diffraction Gemini diffractometer
Absorption correction: analytical (de Meulenaer & Tompa, 1965 ▶) *T*_min_ = 0.723, *T*_max_ = 0.869
4738 measured reflections
4020 independent reflections
3361 reflections with *I* > 2σ(*I*)
*R*_int_ = 0.019

#### Refinement  

*R*[*F* ^2^ > 2σ(*F* ^2^)] = 0.037
*wR*(*F* ^2^) = 0.097
*S* = 1.05
4020 reflections
215 parameters
H atoms treated by a mixture of independent and constrained refinement
Δρ_max_ = 0.33 e Å^−3^
Δρ_min_ = −0.38 e Å^−3^

### 

Data collection: *GEMINI* (Oxford Diffraction, 2006 ▶); cell refinement: *CrysAlis RED* (Oxford Diffraction, 2006 ▶); data reduction: *CrysAlis RED*; program(s) used to solve structure: *SIR2004* (Burla *et al.*, 2005 ▶); program(s) used to refine structure: *SHELXL2013* (Sheldrick, 2008 ▶); molecular graphics: *ORTEP-3 for Windows* (Farrugia, 2012 ▶) and *PLATON* (Spek, 2009 ▶); software used to prepare material for publication: *WinGX* (Farrugia, 2012 ▶).

## Supplementary Material

Crystal structure: contains datablock(s) I. DOI: 10.1107/S160053681401068X/nc2325sup1.cif

Structure factors: contains datablock(s) I. DOI: 10.1107/S160053681401068X/nc2325Isup2.hkl

CCDC reference: 1002145

Additional supporting information:  crystallographic information; 3D view; checkCIF report

## Figures and Tables

**Table 1:** Hydrogen-bond geometry (Å, °)

*D*—H⋯*A*	*D*—H	H⋯*A*	*D*⋯*A*	*D*—H⋯*A*
N1—H1*NA*⋯O2^i^	0.97	2.09	3.022 (3)	162
O1*W*—H1*W*⋯O1	0.85	2.07	2.816 (2)	145
N1—H1*NB*⋯O1	0.97	2.41	3.219 (2)	140
O1*W*—H2*W*⋯N11^ii^	0.95	1.92	2.858 (2)	171
N2—H2*NA*⋯O2^ii^	0.97	2.33	3.189 (3)	147
N2—H2*NA*⋯N11^ii^	0.97	2.47	3.319 (3)	145
N2—H2*NB*⋯O2^iii^	0.97	2.42	3.277 (3)	147
N14—H14*A*⋯N12^iv^	0.93	2.09	3.003 (3)	166
N14—H14*B*⋯O1^v^	0.95	2.21	2.993 (3)	140
N14—H14*B*⋯N13^v^	0.95	2.44	3.215 (3)	139

Symmetry codes: (i) 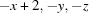; (ii) 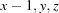; (iii) 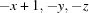; (iv) 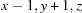; (v) 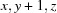.

**Fig. 1.**
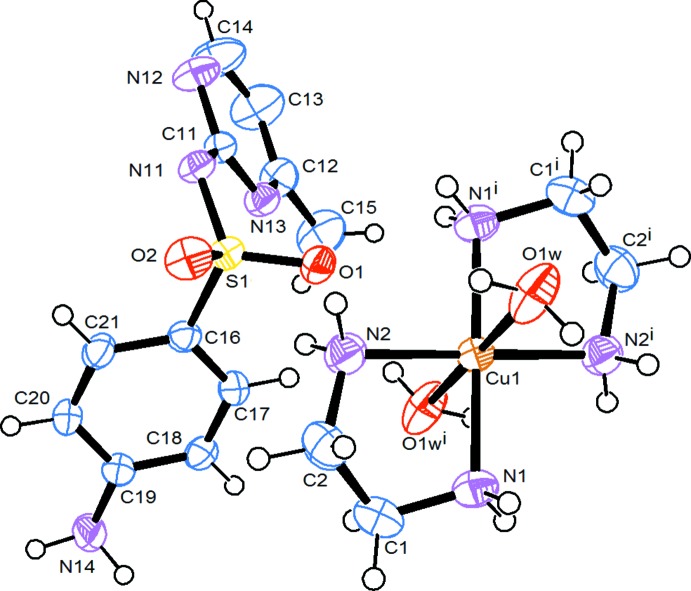
Crystal structure of the title compound with labeling and displacement ellipsoids drawn at the 50% probability level.

**Fig. 2.**
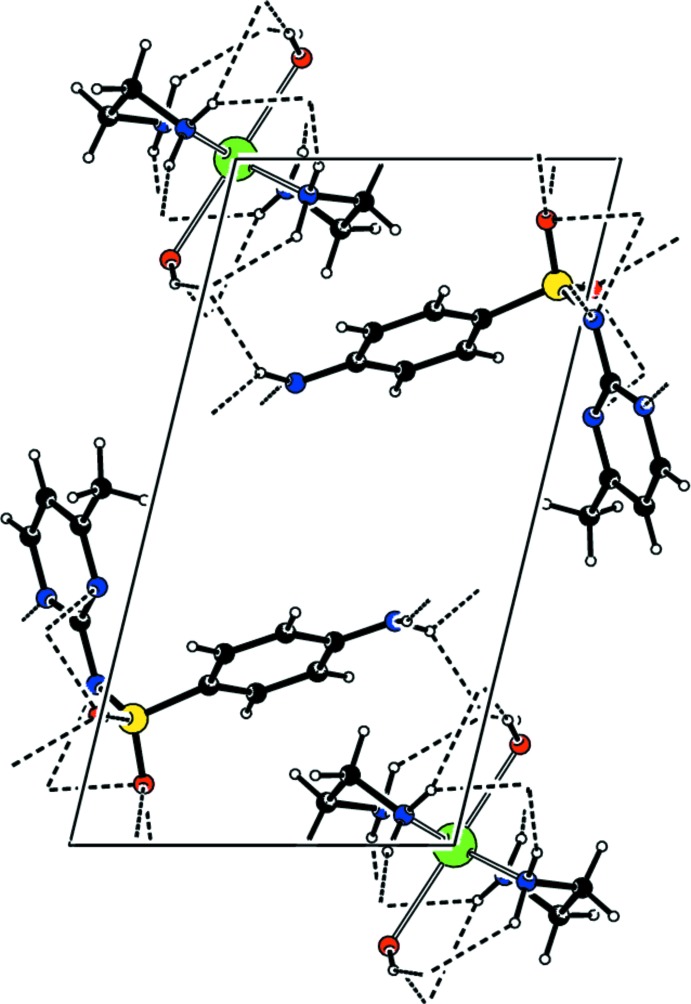
Crystal packing of the title compound, shown through the a axis, with hydrogen bonding drawn as dashed lines.

## supplementary crystallographic information

### 1. Comment

It is well known that many sulfonamide derivatives possess antibacterial activity (Anand, 1980; Kratz *et al.*, 2000; Grave *et al.*, 2010) including sulfamerazine which is widely used to treat susceptible microbial infections (Murphy *et al.*, 1943; Clark *et al.*, 1943). Faster absorption, low overall excretion rate by the kidneys, and an equal therapeutic and toxic effect of sulfamerazine compared to other sulfonamide drugs, such as sulfadiazine and sulfathiazole, have been claimed for its wider usage (Earle, 1944; Forbes *et al.*, 1946).

The first crystal structure of sulfamerazine have been reported by (Acharya *et al.*, 1982). The presence of several potential donor sites, namely the amino, pyrimidine and sulfonamide N atoms and the sulfonyl O atoms, make this ligand a versatile complexing agent.

As part of our efforts to investigate metal(II) complexes based on sulfonamides, we report herein the crystal structure of the new copper(II) complex: Diaquabis(ethylenediamine-κ2 N,*N*')copper(II) bis(sulfamerazinate).

The asymmetric unit of the title compound contains one sulfamerazinate counter-ion and a half [Cu(en)_2_(H_2_O)_2_]^2+^ cation (en = ethylenediamine) (Fig. 1). The metal ion is located in a ML6 environment and coordinated by four N atoms of two ethylenediamine ligands and two O atoms of two water molecules. The four N atoms of the ligands in the equatorial plane form a square-planar arrangement, while the 6-fold coordination is completed by the two water O atoms in the axial positions. The apical Cu—O bridging separation is 2.513 (2) Å while the equatorial Cu—N bridging bond lengths are 2.0168 (19) Å and 2.0016 (18) Å, which is typical for a Jahn-Teller distortion. A sulfamerazine anion that is deprotonated at the N11 N atom is present in the structure (Fig. 1). To the best of our knowledge, a search in the Cambridge Structural Database reveal, that this is the first crystal structure where a sulfamerazinate anion act as a counter ion. A similar situation is observed in [Cu(en)_2_(OH_2_)_2_](Stz)_2_·2H_2_O (Anacona *et al.*, 2002) where a sulfathiazole acts as a deprotonated counter ion too.

In the crystal strcuture of the title compound every complex cation is linked *via* O—H···N, N—H···O and O—H···O hydrogen bonding to the counter cations, while the cations are interconnected *via* the N—H···N interaction, which lead to the formation of a two dimensional network (Fig. 2).

### 2. Experimental

The single crystals of [Cu(C_2_H_8_N_2_)_2_(H_2_O)_2_](C_11_H_12_N_4_SO_2_)_2_ were formed in a methanolic solution (50 ml) of Cu(CH_3_CO_2_)_2_ (0.362 g, 2 mmol) and sulfamerazine (1.057 g, 4 mmol) by adding ethylenediamine (0.5 ml, 7.3 mmol). The precipitate obtained immediately was filtred out and the resulting filtrate was left to slowly evaporate at room temperature which lead to the formation of blue single crystals suitable for X-Ray diffraction.

### 3. Refinement

All C-H and all N-H H atoms of the ethylenediamine molecules were positioned with idealized geometry (methyl H atoms allowed to rotate but not to tip) and were refined isotropic with [*U*_iso_(H) = 1.2 *U*_eq_ (C,*N*)] (1.5 for methyl H atoms) using a riding odel with C—H = 0.93, 0.96 and 0.97 Å (for aromatic, methyl and methylene H atoms and N—H = 0.97 Å for amino H atoms. The N-H and the O-H water H atoms were located in difference map and refined with varying coordinates and fixed isotropic displacement parameters.

### Crystal data

**Table:**

[Cu(C_2_H_8_N_2_)_2_(H_2_O)_2_](C_11_H_11_N_4_O_2_S)_2_	*Z* = 1
*M**_r_* = 746.41	*F*(000) = 391
Triclinic, *P*1	*D*_x_ = 1.431 Mg m^−^^3^
Hall symbol: -P 1	Mo *K*α radiation, λ = 0.71069 Å
*a* = 7.5429 (4) Å	Cell parameters from 3709 reflections
*b* = 8.1800 (5) Å	θ = 3–29°
*c* = 14.8434 (8) Å	µ = 0.81 mm^−^^1^
α = 75.299 (5)°	*T* = 293 K
β = 82.800 (5)°	Block, blue
γ = 78.873 (5)°	0.41 × 0.36 × 0.17 mm
*V* = 866.40 (9) Å^3^	

### Data collection

**Table:**

Oxford Diffraction Gemini diffractometer	4020 independent reflections
Radiation source: fine-focus sealed tube	3361 reflections with *I* > 2σ(*I*)
Graphite monochromator	*R*_int_ = 0.019
ω/2θ scans	θ_max_ = 29.3°, θ_min_ = 2.9°
Absorption correction: analytical (de Meulenaer & Tompa, 1965)	*h* = −10→7
*T*_min_ = 0.723, *T*_max_ = 0.869	*k* = −9→11
4738 measured reflections	*l* = −20→18

### Refinement

**Table:**

Refinement on *F*^2^	Primary atom site location: structure-invariant direct methods
Least-squares matrix: full	Secondary atom site location: difference Fourier map
*R*[*F*^2^ > 2σ(*F*^2^)] = 0.037	H atoms treated by a mixture of independent and constrained refinement
*wR*(*F*^2^) = 0.097	*w* = 1/[σ^2^(*F*_o_^2^) + (0.0359*P*)^2^ + 0.4528*P*] where *P* = (*F*_o_^2^ + 2*F*_c_^2^)/3
*S* = 1.05	(Δ/σ)_max_ < 0.001
4020 reflections	Δρ_max_ = 0.33 e Å^−^^3^
215 parameters	Δρ_min_ = −0.38 e Å^−^^3^
0 restraints	

### Special details

**Table:**

Experimental. The crystal was placed in the cold stream of an Oxford Cryosystems open-flow nitrogen cryostat (Cosier & Glazer, 1986) with a nominal stability of 0.1 K.Cosier, J. & Glazer, A·*M*., 1986. J. Appl. Cryst. 105–107.
Geometry. All e.s.d.'s (except the e.s.d. in the dihedral angle between two l.s. planes) are estimated using the full covariance matrix. The cell e.s.d.'s are taken into account individually in the estimation of e.s.d.'s in distances, angles and torsion angles; correlations between e.s.d.'s in cell parameters are only used when they are defined by crystal symmetry. An approximate (isotropic) treatment of cell e.s.d.'s is used for estimating e.s.d.'s involving l.s. planes.
Refinement. Refinement of *F*^2^ against ALL reflections. The weighted *R*-factor *wR* and goodness of fit *S* are based on *F*^2^, conventional *R*-factors *R* are based on *F*, with *F* set to zero for negative *F*^2^. The threshold expression of *F*^2^ > σ(*F*^2^) is used only for calculating *R*-factors(gt) *etc*. and is not relevant to the choice of reflections for refinement. *R*-factors based on *F*^2^ are statistically about twice as large as those based on *F*, and *R*- factors based on ALL data will be even larger.

### Fractional atomic coordinates and isotropic or equivalent isotropic displacement parameters (Å^2^)

**Table:**

	*x*	*y*	*z*	*U*_iso_*/*U*_eq_	
Cu1	0.5	0	0	0.03130 (12)	
S1	0.93674 (7)	0.08503 (7)	0.18420 (3)	0.03012 (13)	
O1	0.7864 (2)	−0.0053 (2)	0.18937 (11)	0.0387 (4)	
O2	1.0053 (2)	0.1546 (2)	0.08903 (10)	0.0428 (4)	
C20	0.8897 (3)	0.5351 (3)	0.25274 (16)	0.0355 (5)	
H20	0.9615	0.6189	0.2451	0.043*	
N13	0.9256 (2)	−0.0906 (2)	0.37617 (12)	0.0347 (4)	
C21	0.9576 (3)	0.3893 (3)	0.22151 (15)	0.0346 (5)	
H21	1.0743	0.3759	0.1924	0.041*	
C16	0.8532 (3)	0.2613 (3)	0.23311 (14)	0.0291 (4)	
O1W	0.4227 (2)	0.1071 (3)	0.14692 (12)	0.0552 (5)	
C12	0.9149 (3)	−0.1728 (3)	0.46614 (16)	0.0435 (6)	
C11	1.0858 (3)	−0.1138 (3)	0.32650 (15)	0.0310 (4)	
N11	1.1063 (2)	−0.0306 (2)	0.23427 (12)	0.0325 (4)	
N12	1.2372 (3)	−0.2157 (3)	0.36031 (14)	0.0469 (5)	
C15	0.7356 (4)	−0.1427 (5)	0.5202 (2)	0.0659 (8)	
H15A	0.6836	−0.0235	0.5029	0.099*	
H15B	0.752	−0.1751	0.5858	0.099*	
H15C	0.656	−0.2102	0.5066	0.099*	
C13	1.0634 (4)	−0.2794 (4)	0.5060 (2)	0.0652 (9)	
H13	1.0572	−0.3371	0.5686	0.078*	
C14	1.2192 (4)	−0.2967 (4)	0.4501 (2)	0.0658 (9)	
H14	1.3198	−0.3699	0.4761	0.079*	
C17	0.6811 (3)	0.2811 (3)	0.27905 (14)	0.0323 (5)	
H17	0.6123	0.1942	0.2895	0.039*	
N14	0.6421 (3)	0.7043 (3)	0.32650 (17)	0.0488 (5)	
C19	0.7133 (3)	0.5595 (3)	0.29608 (15)	0.0328 (5)	
C18	0.6118 (3)	0.4279 (3)	0.30924 (15)	0.0343 (5)	
H18	0.4955	0.44	0.3389	0.041*	
N1	0.6743 (3)	−0.2106 (2)	0.05258 (13)	0.0382 (4)	
H1NA	0.7839	−0.2193	0.011	0.046*	
H1NB	0.7067	−0.2059	0.113	0.046*	
N2	0.3086 (3)	−0.1504 (2)	0.04393 (14)	0.0394 (4)	
H2NA	0.2099	−0.0953	0.0802	0.047*	
H2NB	0.2601	−0.1696	−0.0093	0.047*	
C1	0.5849 (4)	−0.3587 (3)	0.06228 (18)	0.0455 (6)	
H1A	0.6484	−0.4586	0.1035	0.055*	
H1B	0.5857	−0.384	0.0018	0.055*	
C2	0.3928 (4)	−0.3160 (3)	0.10211 (18)	0.0476 (6)	
H2A	0.3254	−0.4055	0.1017	0.057*	
H2B	0.3916	−0.3071	0.1661	0.057*	
H14A	0.5169	0.7352	0.3264	0.05*	
H14B	0.7011	0.801	0.3119	0.05*	
H1W	0.5101	0.0795	0.182	0.05*	
H2W	0.3232	0.0599	0.182	0.05*	

### Atomic displacement parameters (Å^2^)

**Table:**

	*U*^11^	*U*^22^	*U*^33^	*U*^12^	*U*^13^	*U*^23^
Cu1	0.0291 (2)	0.0302 (2)	0.0334 (2)	−0.00225 (15)	−0.00634 (15)	−0.00569 (15)
S1	0.0257 (3)	0.0345 (3)	0.0295 (3)	−0.0024 (2)	−0.0030 (2)	−0.0079 (2)
O1	0.0305 (8)	0.0411 (9)	0.0488 (9)	−0.0061 (7)	−0.0087 (7)	−0.0156 (7)
O2	0.0432 (9)	0.0525 (10)	0.0277 (8)	−0.0025 (8)	0.0009 (7)	−0.0066 (7)
C20	0.0292 (11)	0.0323 (12)	0.0452 (12)	−0.0109 (9)	−0.0030 (9)	−0.0056 (10)
N13	0.0300 (9)	0.0369 (10)	0.0354 (10)	−0.0041 (8)	−0.0007 (8)	−0.0071 (8)
C21	0.0246 (10)	0.0390 (12)	0.0381 (12)	−0.0075 (9)	0.0004 (9)	−0.0051 (9)
C16	0.0275 (10)	0.0282 (10)	0.0294 (10)	−0.0025 (8)	−0.0047 (8)	−0.0035 (8)
O1W	0.0365 (9)	0.0874 (14)	0.0414 (10)	−0.0189 (9)	−0.0090 (7)	−0.0056 (9)
C12	0.0428 (13)	0.0493 (15)	0.0367 (12)	−0.0103 (11)	0.0000 (10)	−0.0068 (11)
C11	0.0270 (10)	0.0300 (11)	0.0366 (11)	−0.0028 (8)	−0.0029 (8)	−0.0099 (9)
N11	0.0231 (9)	0.0370 (10)	0.0342 (9)	−0.0004 (7)	−0.0007 (7)	−0.0066 (8)
N12	0.0329 (10)	0.0558 (13)	0.0437 (12)	0.0082 (9)	−0.0079 (9)	−0.0061 (10)
C15	0.0537 (17)	0.087 (2)	0.0487 (16)	−0.0136 (16)	0.0144 (13)	−0.0092 (15)
C13	0.0594 (18)	0.082 (2)	0.0379 (14)	0.0001 (16)	−0.0060 (13)	0.0082 (14)
C14	0.0506 (17)	0.078 (2)	0.0508 (17)	0.0136 (15)	−0.0142 (13)	0.0038 (15)
C17	0.0303 (11)	0.0299 (11)	0.0362 (11)	−0.0091 (9)	−0.0002 (9)	−0.0053 (9)
N14	0.0372 (11)	0.0357 (11)	0.0781 (15)	−0.0080 (9)	0.0029 (10)	−0.0239 (10)
C19	0.0316 (11)	0.0300 (11)	0.0357 (11)	−0.0049 (9)	−0.0051 (9)	−0.0050 (9)
C18	0.0252 (10)	0.0361 (12)	0.0397 (12)	−0.0047 (9)	0.0017 (9)	−0.0075 (9)
N1	0.0359 (10)	0.0388 (11)	0.0353 (10)	0.0005 (8)	−0.0066 (8)	−0.0037 (8)
N2	0.0352 (10)	0.0417 (11)	0.0429 (11)	−0.0046 (8)	−0.0037 (8)	−0.0141 (9)
C1	0.0549 (15)	0.0320 (12)	0.0446 (14)	−0.0010 (11)	−0.0043 (11)	−0.0047 (10)
C2	0.0555 (16)	0.0379 (14)	0.0468 (14)	−0.0136 (12)	0.0009 (12)	−0.0034 (11)

### Geometric parameters (Å, º)

**Table:**

Cu1—N1	2.0016 (18)	C15—H15B	0.96
Cu1—N1^i^	2.0016 (18)	C15—H15C	0.96
Cu1—N2	2.0168 (19)	C13—C14	1.357 (4)
Cu1—N2^i^	2.0168 (19)	C13—H13	0.93
S1—O2	1.4524 (16)	C14—H14	0.93
S1—O1	1.4530 (16)	C17—C18	1.374 (3)
S1—N11	1.5806 (17)	C17—H17	0.93
S1—C16	1.752 (2)	N14—C19	1.364 (3)
C20—C21	1.373 (3)	N14—H14A	0.9300
C20—C19	1.404 (3)	N14—H14B	0.9500
C20—H20	0.93	C19—C18	1.399 (3)
N13—C12	1.334 (3)	C18—H18	0.93
N13—C11	1.341 (3)	N1—C1	1.465 (3)
C21—C16	1.393 (3)	N1—H1NA	0.97
C21—H21	0.93	N1—H1NB	0.97
C16—C17	1.389 (3)	N2—C2	1.481 (3)
O1W—H1W	0.8500	N2—H2NA	0.97
O1W—H2W	0.9500	N2—H2NB	0.97
C12—C13	1.376 (4)	C1—C2	1.505 (4)
C12—C15	1.494 (4)	C1—H1A	0.97
C11—N12	1.347 (3)	C1—H1B	0.97
C11—N11	1.370 (3)	C2—H2A	0.97
N12—C14	1.332 (3)	C2—H2B	0.97
C15—H15A	0.96		
			
N1—Cu1—N1^i^	180	N12—C14—C13	124.2 (2)
N1—Cu1—N2	85.23 (8)	N12—C14—H14	117.9
N1^i^—Cu1—N2	94.77 (8)	C13—C14—H14	117.9
N1—Cu1—N2^i^	94.77 (8)	C18—C17—C16	120.5 (2)
N1^i^—Cu1—N2^i^	85.23 (8)	C18—C17—H17	119.7
N2—Cu1—N2^i^	180	C16—C17—H17	119.7
O2—S1—O1	113.23 (10)	C19—N14—H14A	115.00
O2—S1—N11	105.46 (9)	C19—N14—H14B	122.00
O1—S1—N11	113.64 (10)	H14A—N14—H14B	112.00
O2—S1—C16	106.20 (10)	N14—C19—C18	120.2 (2)
O1—S1—C16	106.87 (10)	N14—C19—C20	122.0 (2)
N11—S1—C16	111.27 (10)	C18—C19—C20	117.8 (2)
C21—C20—C19	121.0 (2)	C17—C18—C19	121.1 (2)
C21—C20—H20	119.5	C17—C18—H18	119.5
C19—C20—H20	119.5	C19—C18—H18	119.5
C12—N13—C11	117.89 (19)	C1—N1—Cu1	107.55 (14)
C20—C21—C16	120.5 (2)	C1—N1—H1NA	110.2
C20—C21—H21	119.7	Cu1—N1—H1NA	110.2
C16—C21—H21	119.7	C1—N1—H1NB	110.2
C17—C16—C21	119.0 (2)	Cu1—N1—H1NB	110.2
C17—C16—S1	121.54 (16)	H1NA—N1—H1NB	108.5
C21—C16—S1	119.33 (16)	C2—N2—Cu1	108.34 (14)
H1W—O1W—H2W	107.00	C2—N2—H2NA	110
N13—C12—C13	120.8 (2)	Cu1—N2—H2NA	110
N13—C12—C15	116.8 (2)	C2—N2—H2NB	110
C13—C12—C15	122.4 (2)	Cu1—N2—H2NB	110
N13—C11—N12	124.9 (2)	H2NA—N2—H2NB	108.4
N13—C11—N11	120.69 (18)	N1—C1—C2	108.2 (2)
N12—C11—N11	114.39 (19)	N1—C1—H1A	110.1
C11—N11—S1	119.66 (14)	C2—C1—H1A	110.1
C14—N12—C11	115.0 (2)	N1—C1—H1B	110.1
C12—C15—H15A	109.5	C2—C1—H1B	110.1
C12—C15—H15B	109.5	H1A—C1—H1B	108.4
H15A—C15—H15B	109.5	N2—C2—C1	108.23 (19)
C12—C15—H15C	109.5	N2—C2—H2A	110.1
H15A—C15—H15C	109.5	C1—C2—H2A	110.1
H15B—C15—H15C	109.5	N2—C2—H2B	110.1
C14—C13—C12	117.2 (2)	C1—C2—H2B	110.1
C14—C13—H13	121.4	H2A—C2—H2B	108.4
C12—C13—H13	121.4		
			
C19—C20—C21—C16	−0.7 (3)	C16—S1—N11—C11	64.56 (19)
C20—C21—C16—C17	−1.8 (3)	N13—C11—N12—C14	0.1 (4)
C20—C21—C16—S1	174.88 (16)	N11—C11—N12—C14	179.7 (2)
O2—S1—C16—C17	129.97 (17)	N13—C12—C13—C14	0.0 (5)
O1—S1—C16—C17	8.82 (19)	C15—C12—C13—C14	−179.9 (3)
N11—S1—C16—C17	−115.76 (17)	C11—N12—C14—C13	−1.0 (5)
O2—S1—C16—C21	−46.61 (18)	C12—C13—C14—N12	1.0 (5)
O1—S1—C16—C21	−167.75 (16)	C21—C16—C17—C18	2.7 (3)
N11—S1—C16—C21	67.66 (18)	S1—C16—C17—C18	−173.90 (16)
C11—N13—C12—C13	−0.8 (4)	C21—C20—C19—N14	−179.1 (2)
C11—N13—C12—C15	179.1 (2)	C21—C20—C19—C18	2.2 (3)
C12—N13—C11—N12	0.8 (3)	C16—C17—C18—C19	−1.1 (3)
C12—N13—C11—N11	−178.8 (2)	N14—C19—C18—C17	180.0 (2)
N13—C11—N11—S1	−3.7 (3)	C20—C19—C18—C17	−1.3 (3)
N12—C11—N11—S1	176.63 (17)	Cu1—N1—C1—C2	−43.1 (2)
O2—S1—N11—C11	179.29 (17)	Cu1—N2—C2—C1	−35.0 (2)
O1—S1—N11—C11	−56.1 (2)	N1—C1—C2—N2	52.3 (3)

Symmetry code: (i) −*x*+1, −*y*, −*z*.

### Hydrogen-bond geometry (Å, º)

**Table:**

*D*—H···*A*	*D*—H	H···*A*	*D*···*A*	*D*—H···*A*
N1—H1*NA*···O2^ii^	0.97	2.09	3.022 (3)	162
O1*W*—H1*W*···O1	0.85	2.07	2.816 (2)	145
N1—H1*NB*···O1	0.97	2.41	3.219 (2)	140
O1*W*—H2*W*···N11^iii^	0.95	1.92	2.858 (2)	171
N2—H2*NA*···O2^iii^	0.97	2.33	3.189 (3)	147
N2—H2*NA*···N11^iii^	0.97	2.47	3.319 (3)	145
N2—H2*NB*···O2^i^	0.97	2.42	3.277 (3)	147
N14—H14*A*···N12^iv^	0.93	2.09	3.003 (3)	166
N14—H14*B*···O1^v^	0.95	2.21	2.993 (3)	140
N14—H14*B*···N13^v^	0.95	2.44	3.215 (3)	139
C17—H17···O1	0.93	2.55	2.915 (3)	104

Symmetry codes: (i) −*x*+1, −*y*, −*z*; (ii) −*x*+2, −*y*, −*z*; (iii) *x*−1, *y*, *z*; (iv) *x*−1, *y*+1, *z*; (v) *x*, *y*+1, *z*.
